# Complete Genome Sequence of Cyanobacterium *Leptolyngbya* sp. NIES-3755

**DOI:** 10.1128/genomeA.00090-16

**Published:** 2016-03-17

**Authors:** Yuu Hirose, Takatomo Fujisawa, Yoshiyuki Ohtsubo, Mitsunori Katayama, Naomi Misawa, Sachiko Wakazuki, Yohei Shimura, Yasukazu Nakamura, Masanobu Kawachi, Hirofumi Yoshikawa, Toshihiko Eki, Yu Kanesaki

**Affiliations:** a Department of Environmental and Life Sciences, Toyohashi University of Technology, Tempaku, Toyohashi, Aichi, Japan; b Electronics-Inspired Interdisciplinary Research Institute (EIIRIS), Toyohashi University of Technology, Tempaku, Toyohashi, Aichi, Japan; c Center for Information Biology, National Institute of Genetics, Research Organization of Information and Systems, Yata, Mishima, Japan; d Department of Environmental Life Sciences, Graduate School of Life Sciences, Tohoku University, Sendai, Miyagi, Japan; e College of Industrial Technology, Nihon University, Narashino, Chiba, Japan; f Center for Environmental Biology and Ecosystem Studies, National Institute for Environmental Studies, Tsukuba, Ibaraki, Japan; g Department of Bioscience, Tokyo University of Agriculture, Setagaya-ku, Tokyo, Japan; h Genome Research Center, Tokyo University of Agriculture, Setagaya-ku, Tokyo, Japan

## Abstract

Cyanobacterial genus *Leptolyngbya* comprises genetically diverse species, but the availability of their complete genome information is limited. Here, we isolated *Leptolyngbya* sp. strain NIES-3755 from soil at the Toyohashi University of Technology, Japan. We determined the complete genome sequence of the NIES-3755 strain, which is composed of one chromosome and three plasmids.

## GENOME ANNOUNCEMENT

Cyanobacteria of genus *Leptolyngbya* have thin filaments (0.5 to 3.5 µm wide) and parietal location of thylakoids. Genetically diverse *Leptolyngbya* have been isolated from various environments (1, 2), but the availability of high-quality genome information is limited. To understand the molecular diversity of the genus *Leptolyngbya*, we isolated *Leptolyngbya* sp. strain NIES-3755 from soil at the Toyohashi University of Technology, Toyohashi, Aichi, Japan, and determined the complete genome sequence.

Whole-genome sequencing of the NIES-3755 strain was performed using MiSeq sequencer (Illumina, San Diego, CA) and *in silico* finishing software as reported previously (3–6). An 800-bp paired-end library and an 8-kbp mate-pair library were prepared using the TruSeq DNA PCR-free sample preparation kit (Illumina) and Nextera mate-pair sample preparation kit (Illumina), respectively. 300 bp of each end of the libraries were sequenced on the MiSeq instrument with the MiSeq reagent kit v3 (600-cycles; Illumina). The reads were filtered using ShortReadManager based on 17-mer frequency (7). A total of 263 Mbp of paired-end reads and 50 Mbp of mate-pair reads were assembled using Newbler version 2.9 (Roche), yielding 7 scaffolds and 118 large contigs (>500 bp). Gap sequences between the contigs were first determined *in silico* using GenoFinisher and AceFileViewer (7). Two gap sequences containing complicated repeats were determined by Sanger sequencing. We determined the complete genome sequence of *Leptolyngbya sp*. NIES-3755, which comprises one chromosome and three plasmids (total 6,761,657 bp). The G+C content of the genome was calculated to be 46.7%. A total of 5,897 protein-encoding genes, 6 rRNA genes, and 63 tRNA genes were predicted using the Prokka software (8). Annotation was performed via original scripts that refer to the manually curated annotation in Cyanobase (9, 10). Within cyanobacterial genome sequences in the NCBI database, the NIES-3755 strain is most closely but distantly related to *Leptolyngbya boryana* dg5 with 95.2% of 16S rRNA identity (11). The high-quality genome information of the NIES-3755 strain will facilitate our understanding of the genetic diversity of the *Leptolyngbya* genus.

### Nucleotide sequence accession numbers.

The complete genome sequence of *Leptolyngbya* sp*.* NIES-3755 has been deposited in the DNA Data Bank of Japan under accession numbers AP017308 to AP017311. The NIES-3755 strain is available at the Microbial Culture Collection at the National Institute for Environmental Studies (NIES) in Japan (http://mcc.nies.go.jp/).
